# ASSESSING MOBILITY AND FALL RISKS IN TAIWAN’S OLDER ADULT POPULATION: A LONGITUDINAL STUDY

**DOI:** 10.1093/geroni/igae098.3361

**Published:** 2024-12-31

**Authors:** Fang-Lin Kuo, Zih-Yong Liao

**Affiliations:** National Health Research Institutes, Yunlin, Yunlin, Taiwan (Republic of China); National Yang Ming Chiao Tung University, Taipei, Taiwan (Republic of China)

## Abstract

Mobility difficulties impact older adults’ health significantly, leading to adverse outcomes and loss of independence. In Taiwan, 21% of community-dwelling older adults report falls, and over half have a fear of falling, highlighting the importance of identifying individuals at risk. This study aimed to explore mobility changes and repeated falls in Taiwan’s rural areas, where the older population exceeds 24%. A longitudinal study was conducted annually from 2022 to 2023, focusing on physical ability and gait assessments in community-dwelling older adults (65+) who were able to walk (n=250). We used linear mixed-effects regression models and logit models to examine whether personal characteristics are associated with changes in mobility and falls. The findings showed no significant differences in falls and walking performance at the 1-year follow-up after confounder adjustments. However, individuals living in buildings without elevators, using walking devices with cognitive impairment and exhibiting poorer gait performance such as slower gait speed, peak angle velocity at swing phase and toe clearance were more likely to experience repeated falls. Furthermore, about 14.78% of participants who were independently walking at baseline began using a walking device by 2023, indicating a transition to new walking dependence (NWD). The NWD group tended to have a higher number of chronic illnesses, repeated falls and slower walking speed. This study highlights the need to consider environmental and gait characteristics in addressing the mobility dependence and fall risks. Interventions to prevent falls and facilitate the adaptation to walking assistive devices could help manage mobility decline among older adults.

